# Spatial-Temporal Characteristics of Agriculture Green Total Factor Productivity in China, 1998–2016: Based on More Sophisticated Calculations of Carbon Emissions

**DOI:** 10.3390/ijerph16203932

**Published:** 2019-10-16

**Authors:** Xiaocang Xu, Xiuquan Huang, Jun Huang, Xin Gao, Linhong Chen

**Affiliations:** 1 Research Center for Economy of Upper Reaches of the Yangtse River/School of Economics, Chongqing Technology and Business University, Chongqing 400067, China; cangxiaoxu@ctbu.edu.cn; 2 Background Operation Center of Credit Card, China Minsheng Bank (Chengdu), Chengdu 610100, China; lingyunchuangxin@163.com; 3 Business School, Hohai University, Nanjing 211100, China; gxtz1987@hhu.edu.cn; 4 School of Mathematics and Statistics, Chongqing Technology and Business University, Chongqing 400067, China; 2017325010005@stu.scu.edu.cn; 5 School of Public Administration, Sichuan University, Chengdu 610065, China

**Keywords:** green total factor productivity, agricultural carbon emissions, spatio-temporal differentiation, spatial correlation, time evolution, carbon sources

## Abstract

Environmental costs should be taken into account when measuring the achievements of China’s agricultural development, since the long-term extensive development of agriculture has caused huge environmental pollution. This study took agricultural carbon emissions as an undesired output to estimate the agricultural development efficiency in 31 provinces of China from 1998 to 2016, based on the green total factor productivity, as assessed by the slacks-based measure directional distance function and constructing the global Malmquist–Luenberger index. We measured agricultural carbon emissions in terms of five aspects: agricultural materials, rice planting, soil, livestock and poultry farming, and straw burning, and then compared the green total factor productivity index and the total factor productivity index. The study came to the following conclusions: (1) the green technology efficiency change was smaller than the technology efficiency change at first, but the gap between them is narrowing with time, such that the former is now larger than the latter; (2) the green technology efficiency was in a declining state and the green technology progress was increasing, promoting the green total factor productivity growth, from 1998 to 2016; and (3) China’s agricultural green total factor productivity increased by 4.2% annually in the east, 3.4% annually in the central region, and 2.5% annually in the west.

## 1. Introduction

Agriculture is a key sector in China, providing the most basic goods to meet people’s basic needs in order to keep the economy running, and the Chinese government also attaches great importance to the development of agriculture. China’s agriculture has developed rapidly in recent years and achieved great progress [1]. In 2017, China’s total agriculture output value (including crop, livestock, forestry, and fishery) reached 6.8 billion yuan, with 134.9 million hectares of arable land and 361.8 million agricultural practitioners [2]. However, the development of agriculture in China has caused serious pressure on the environment [3], because it produces a large amount of carbon emissions each year, accounting for a high proportion of all carbon emissions—much higher than the United States and the world average [4,5,6,7]. Greenhouse gas emissions (including CO_2_, CH_4_, N_2_O, perfluorocarbons, hydrofluorocarbons, etc.) are often measured by carbon emissions and will threaten human survival and development. Therefore, we should take environmental pollution into account to comprehensively measure the development of China’s agriculture [8].

In fact, many international organizations have begun to attach importance to the relationship between agricultural development and environmental pollution and put forward some relevant measurement frameworks for the development level of green agriculture and suggestions for promoting the development of green agriculture. The meaningful studies in this area include *Green Growth Indicators for Agriculture: A Preliminary Assessment* by the Organization for Economic Co-operation and Development (2014) [9].

Academia generally measures development in terms of productivity. Productivity refers to the utilization efficiency of human, material and financial resources, which reflects the influence of resource allocation, technological level, and labor force on production activities. From the initial single factor, productivity has evolved into the commonly used total factor productivity (TFP) [10,11]. In the original TFP studies, undesirable outputs such as pollutant emissions were not taken into account. With the deepening of the research, green total factor productivity (GTFP), considering non-expected output, has emerged.

In recent years, there have been many research projects on agricultural TFP, which can be divided into three categories according to the measurement method. The first category is to use the growth accounting method for measurement; this was often used in relatively early studies. Many scholars used the algebraic index method to analyze China’s agricultural productivity [12,13], and many used the Solow residual method [14,15]. These kinds of methods are not outdated, because they are relatively simple and have advantages in some current studies. For example, some scholars make good use of them to calculate TFP with micro data [16,17].

In the second category, data envelopment analysis (DEA) is used to analyze the changes of China’s agricultural productivity. The advantage of this method is that it can deal with multiple input and output variables, and it facilitates the decomposition of agricultural productivity to understand the internal growth momentum of agricultural productivity. DEA has been widely used in empirical analysis [18,19,20]. The third category of research uses stochastic frontier analysis (SFA). According to whether it is necessary to set the specific form of production function in advance, this type of analysis can be further divided into the parametric method and non-parametric method. According to whether the production frontier is affected by random factors, it can also be divided into the stochastic frontier method and deterministic frontier method [21,22]. Similarly, the research on agricultural GTFP can also be divided into four categories according to the measurement method. The first three categories are the same as for agricultural TFPs, and the only difference is that the three methods take environmental pollution as an input variable. The fourth category includes those studies considering pollution as an undesirable output to calculate the GTFP [23,24].

This paper took agricultural carbon emissions (ACEs) as an undesired output to calculate China’s agricultural green total factor productivity. It is difficult to achieve a breakthrough in the research of ACEs in terms of the calculation method. Therefore, more progress tends to be made in the selection of carbon emission sources. West et al. chose agricultural inputs, including fertilizers, agricultural limes, pesticides, agricultural irrigation and seed cultivation, as agricultural carbon emission sources (ACESs) [25]. Johnson et al. argued that ACEs are mainly derived from intestinal fermentation of livestock, manure management, rice growth, and arbitrary disposal of agricultural waste [26]. Other studies, such as those by Tan Q. and Wu Y. et al., determined the sources as having four aspects: rice planting, rice fields, agricultural material input, livestock and poultry farming, and soil management. The index is adequate, but the ACE coefficients were not listed in the paper in detail [27,28].

Like many studies focused on measuring GTFP, this paper takes ACEs as an undesired output. However, a new breakthrough was achieved in making the GTFP more closely reflect reality in the calculation of ACEs. This study calculated ACEs in China by choosing five categories of ACEs: agricultural materials, rice cultivation, N_2_O emissions due to the damage of the soil surface when planting crops, livestock and poultry farming, and crop straw burning. Contrary to other research, the species of rice, crops on the land and livestock and poultry, and the location of rice, were taken into account when calculating ACEs from rice planting, soil N_2_O, and livestock and poultry farming, because carbon emissions of those ACESs could be influenced by these factors. In addition, the study added agriculture straw combustion as one of the ACESs, which improved the estimation accuracy for ACEs.

This paper consists of four parts. The first part and the second are the introduction and method, respectively. Section 3 presents the comparison of green total factor productivity index (GTFPI)and total factor productivity index (TFPI), the temporal and spatial characteristics of GTFPI, and the analysis of the spatial correlation of GTFPI in China. The discussion and conclusion are presented in Section 4.

## 2. Materials and Methods

### 2.1. Measurement Way of Green Total Factor Productivity

#### 2.1.1. Global Production Possibility Set

China’s 31 provinces (excluding Hong Kong, Macao and Taiwan) are all taken as decision-making units. Suppose each unit uses n kinds of input (*x*, *x* ∊ *R*^*n*_+_^) in the production process, and obtains both m kinds of desired output (*y*, *y* ∊ *R*^*m*_+_^) and undesired output—ACEs (*b*). The set of possibilities between inputs, desirable output, and undesired output is defined as environmental production technology [29], which combined with its bounded closure, the strong disposition of input and undesired output, weak disposition of desirable output and undesired output, and zero binding, and the production possible set (*P*) is expressed as Equation (1):

(1) P(x)={(x,y,b):x can produce (y,b)}

Further, according to the global production possibility set proposed by OH [30], the union set of all production technology sets in the current period can be expressed as Equation (2):

(2) $$P^{G}\left(x\right)=P^{1}\left(x^{1}\right)\cup P^{2}\left(x^{2}\right)\ldots P^{T}\left(x^{T}\right)$$

where *G* is the global benchmark and *T* is the period. Under the condition that the remuneration of production technology scale remains unchanged, assume the time period *t* = 1, 2, ..., *T* and decision-making unit *k* = 1, 2, ..., *K*, the input and output vector is $\left(x_{k}^{t}+y_{k}^{t}+b_{k}^{t}\right)$, then the global production possibility set is expressed as:

(3) $$P^{G}\left(x\right)=\left\{\left(x^{t}+y^{t}+b^{t}\right):\overset{T}{\underset{t=1}{\sum }}\overset{K}{\underset{k=1}{\sum }}Z_{k}y_{km}^{t}\geq y_{m}^{t};\overset{T}{\underset{t=1}{\sum }}\overset{K}{\underset{k=1}{\sum }}Z_{k}b_{k}^{t}=b^{t};\overset{T}{\underset{t=1}{\sum }}\overset{K}{\underset{k=1}{\sum }}Z_{k}x_{kn}^{t}\leq x_{n}^{t}\right\}$$

$Z_{k}$ is the density variable, which represents the weight of each of k decision-making units in the construction if the environmental technical structure.

#### 2.1.2. Slacks-Based Measure (SBM) Directional Distance Function

The related efficiency measurement idea and models belong to radial and linear piecewise measurement theory. The main features of this measurement: strong disposable, namely its linear piecewise frontier sometimes parallel to the horizontal axis or the vertical axis, ensures the efficiency boundary or indifference curve convexity (not bending), but also causes input “congestion” or “slacks”. At the same time, the linear piecewise front obtained by linear programming also violates free disposal—the basic assumption of Neoclassical economics.

**Figure 1:**
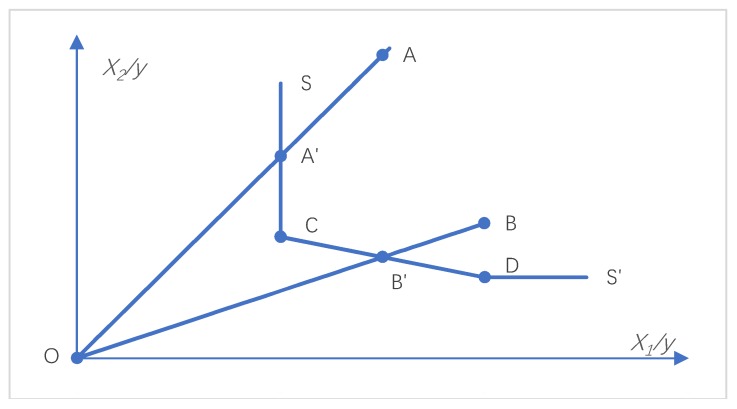
Efficiency measure and input slacks.

Figure 1 illustrates this problem nicely. The frontier of production technology is the linear segment frontier SS’, composed of two efficient production units at point C and point D. The points on the broken line of SS′are the efficient production points of the production technology. Therefore, the production units A and B are the inefficient production points. According to the measurement method of radial technical efficiency, the degree of technical efficiency of A and B can be expressed as OA′/OA and OB′/OB. A′ and B′ on the production front are the efficient reference points of A and B. Like C and D, A′ and B′ are points of technical efficiency 1. Comparing point A′ with point C, we find that the production at point A′ can produce the same output y as the production at point C by continuing to reduce the input of X_2_, and this condition is called input slacks. If the model is extended to multiple inputs and multiple outputs, there will also be output slacks. Complete efficiency requires neither inefficiency nor input slacks, which is a problem that traditional DEA models have been unable to solve.

The SBM directional distance function can consider the influence of input and output slack variables on efficiency, and it can avoid the bias of traditional radial DEA on efficiency evaluation. According to the non-radial and non-angle SBM efficiency model proposed by TONE [31], both input reduction and output increase are considered. On the basis of Equation (3), the non-radial, non-angle SBM directional distance function containing an undesired output in time t of a decision-making unit $k^{\prime }\left(x_{k}^{t}+y_{k}^{t}+b_{k}^{t}\right)$ is constructed:

(4) $${\vec{D}}_{0}^{G}\left(x_{k}^{t}+y_{k}^{t}+b_{k}^{t}\right)=min\frac{1-\left[\frac{1}{N}\sum_{n=1}^{N}s_{n}^{x}/k_{n}^{k^{\prime }}\right]}{1+\left[\frac{1}{M+1}\left(\sum_{m=1}^{M}s_{m}^{y}/y_{m}^{k^{\prime }}+\sum_{i=1}^{I}s_{i}^{b}/b_{i}^{k^{\prime }}\right)\right]}$$

(5) $$x_{k\prime n}^{t}=\overset{T}{\underset{t=1}{\sum }}\overset{K}{\underset{k=1}{\sum }}Z_{k}^{t}x_{kn}^{t}+s_{n}^{x},n=1,2,\ldots ,N$$

(6) $$y_{k^{\prime }m}^{t}=\overset{T}{\underset{t=1}{\sum }}\overset{K}{\underset{k=1}{\sum }}Z_{k}^{t}y_{km}^{t}-s_{m}^{y},m=1,2,\ldots ,M$$

(7) $$b_{k^{\prime }i}^{t}=\overset{T}{\underset{t=1}{\sum }}\overset{K}{\underset{k=1}{\sum }}Z_{k}^{t}b_{ki}^{t}+s_{i}^{b},i=1,2,\ldots ,I$$

In Equations (4)–(7), ${\vec{D}}_{0}^{G}$ is the average distance between input and output and the production front—the inefficiency degree of input and output. $s_{n}^{x}$ $s_{m}^{y}$ and $s_{i}^{b}$ are the relaxation variables of input, desired output, and undesired output, respectively, all of which are greater than or equal to 0. When $s_{n}^{x}$ $=s_{m}^{y}$ = $s_{i}^{b}$ = 0, the decision-making unit is completely efficient and there is no surplus of input or insufficient output. Otherwise, it means that there is an efficiency loss.

#### 2.1.3. Global Malmquist-Luenberger (GML) Index

The GML index is based on the common global frontier structure of each period, which is multiplicative and with transitivity, which can effectively overcome the defects of Malmquist-Luenberger index and avoid the phenomenon of “technical regression”.

In this paper, the GML index was used to construct the green total factor productivity index (GTFPI), which represented changes in TFP. GTFPI could be decomposed into the green technology efficiency change (GTEC) and green technical progress change (GTPC). The three indexes were established as Equations (8)–(11). If the index was greater than 1, it meant that the variables at stage t+1 were higher than that at stage t, and vice versa.

(8) $$GTFPI=\frac{1+{\vec{D}}_{0}^{G}\left(X^{t},y^{t},b^{t};y^{t},-b^{t}\right)}{1+{\vec{D}}_{0}^{G}\left(X^{t+1},y^{t+1},b^{t+1};y^{t+1},-b^{t+1}\right)}$$

(9) GTFPI=GTEC∗GTPC

(10) $$GTEC=\frac{1+{\vec{D}}_{0}^{G}\left(X^{t},y^{t},b^{t};y^{t},-b^{t}\right)}{1+{\vec{D}}_{0}^{t+1}\left(X^{t+1},y^{t+1},b^{t+1};y^{t+1},-b^{t+1}\right)}$$

(11) $$GTPC=\frac{1+{\vec{D}}_{0}^{t+1}\left(X^{t+1},y^{t+1},b^{t+1};y^{t+1},-b^{t+1}\right)}{1+{\vec{D}}_{0}^{G}\left(X^{t+1},y^{t},b^{t+1};y^{t+1},-b^{t+1}\right)}$$

### 2.2. The Selection of Input and Output Indicators

The input and output index system of GTFPI, measured by each province, is shown in Table 1. In this paper, input indexes were selected from eight aspects. In terms of output, the desired output was the actual output value of agriculture, forestry, husbandry and fisheries, calculated for 1997, and ACEs were selected as undesired output indexes. This paper compiled a huge ACE measurement system, which was one of its notable innovations.

### 2.3. Estimation of Agricultural Carbon Emissions

A measurement system was compiled to calculate ACEs. The carbon emission coefficients represented how much carbon was produced per unit by ACES, and they came from the related natural science research. This study calculated ACEs in China by choosing five categories of ACESs: agricultural materials, rice cultivation, N_2_O emissions due to the damage to the soil surface when planting crops, livestock and poultry farming, and crop straw burning, and each of the five categories included various different types of ACES.

The ACESs could be converted into ACEs by the Equation (12):

(12) $$E=\sum E_{i}=\sum Q_{i}\times a_{i}$$

where E represented the total ACEs, $E_{i}$ represented the ACE of the i-th ACES, the quantity of the i-th ACES was represented by $Q_{i}$ and $a_{i}$ represented the carbon emission coefficient of the i-th ACES.

ACESs in this paper emitted CH_4_ and N_2_O gases, and they need be converted to standard carbon emissions before calculating ACEs. According to the Intergovernmental Panel on Climate Change (2007) report [32], in terms of the greenhouse effect, 1 ton of CH_4_ is equivalent to 6.8182 t of C, and one ton of N_2_O is equivalent to 81.2727 t of C. It could be converted according to Equation (13):

(13) $$C=C_{CH_{4}}+C_{N_{2}O}=\sum CH_{4}*6.8182+\sum N_{2}O*81.2727$$

### 2.4. Agricultural Carbon Emission Sources

#### 2.4.1. Carbon Emissions from Agricultural Materials

All research on agricultural carbon emissions considers agricultural materials as one of the ACESs. In fact, in earlier studies, agricultural materials were considered as the only one. According to existing relevant studies [32,33], fertilizer, pesticides, plastic sheeting, diesel oil, and irrigation were considered carbon sources from agricultural materials, and their carbon emission coefficients were 0.8956, 4.9341, 5.18, 0.5927 and 266.48, respectively. The units are all kg C/kg.

#### 2.4.2. Carbon Emissions from Rice Cultivation

Rice cultivation produces CH_4_. China, as the country with the highest yield of rice in the world, can account for a large proportion of its ACEs coming from CH_4_. Other dryland crops can also emit CH_4_ but can partially reabsorb it themselves, so production from other sources was so small that it is ignored in this study. Taking the location and the species of rice into account, the CH_4_ emission coefficients are shown in Table 2 [34].

#### 2.4.3. N_2_O Emissions due to Damage to the Soil Surface When Planting Crops

When crops are planted, the surface of the soil is turned over and large amounts of greenhouse gases are released into the air, most notably N_2_O. In addition, compared with other greenhouse gases, N_2_O gas has a great potential to contribute to temperature increases and a long retention time, with obvious negative effects. Although CO_2_ is also released, the amount is not large and some of the CO_2_ can be absorbed, so it is ignored in this study. Similarly, the variety of crop affects N_2_O emissions, so we considered six types of crops: paddy rice, winter wheat, spring wheat, soybean, corn, and vegetables, and their N_2_O emission coefficients were 0.24, 2.05, 0.4, 0.77, 2.532 and 4.21, respectively [35,36]. All the units are g/m^2^.

#### 2.4.4. Carbon Emissions from Livestock and Poultry Farming

The manure management systems and enteric fermentation during livestock and poultry farming activities produce CH_4_ and N_2_O. Before measuring carbon emissions from livestock and poultry farming, the amount of feeding should be properly adjusted because of the different breeding cycle of each kind of livestock or poultry. This study selected the top 12 species of animals in Chinese agriculture: cows, buffalo, cattle, mules, camels, donkeys, horses, live pigs, sheep, goats, rabbits, and poultry.

Pigs, rabbits and poultry have an average life cycle of 200 days, 105 days and 55 days, respectively, and their slaughter rates are all greater than 1, so their numbers are adjusted according to Equation (14):

(14) $$N_{i}=DL_{i}*\frac{M_{i}}{365}$$

$N_{i}$ represents the average annual feeding amount of livestock or poultry, $DL_{i}$ is the average life cycle, and $M_{i}$ is the annual output.

The slaughter rates of the other selected livestock and poultry are less than 1, so they are adjusted according to Equation (15):

(15) $$N_{i}=\frac{C_{i,t}+C_{i,t-1}}{2}$$

$N_{i}$ is the average annual feeding amount, and $C_{i,t}$ and $C_{i,t-1}$ represent the year-end inventory of years  t and t−1, respectively.

To calculate carbon emissions from livestock and poultry farming, this paper includes 12 kinds of animals that are the most cultivated in Chinese agriculture, and their CH_4_ and N_2_O emission coefficients are listed in Table 3 [33].

#### 2.4.5. Carbon Emissions from Straw Burning

Straw burning, which is very polluting to the environment, has gradually attracted the attention of scholars and is one category of carbon emission sources in this paper. The six main crop straws, rice, wheat, corn, rapeseed, soybean and cotton, were selected as carbon sources of straw burning. According to the existing research [37], the carbon emission coefficients of rice, wheat, corn, rape, soybean and cotton straw are 0.18, 0.16, 0.17, 0.22, 0.15 and 0.13, respectively, and all the units are kg C/kg.

### 2.5. Sample Selection and Data Sources

The sample includes data from 31 provinces in China (excluding Hong Kong, Macao and Taiwan) from 1997 to 2016, and it was obtained from the China Rural Statistical Yearbook, China Agricultural Yearbook, China Agricultural Statistics, China Animal Husbandry Yearbook, and Statistical Yearbook of China.

### 2.6. Process

This paper initially calculated ACEs of 31 provinces in China from 1997 to 2016 through the agricultural carbon emission measurement system. Taking the calculated ACEs as the undesired output, GTFPI was calculated by combining the SBM model with the global Malmquist–Luenberger index, according to the input and output index system. In addition, this paper calculated the traditional agricultural total factor productivity index (TFPI); that is, only the actual agricultural, forestry, husbandry and fishery output values were used as the desired output index, without undesired output.

This paper compared GTFPI and its decomposition terms with TFPI and its decomposition terms to analyze the impact of carbon emissions when calculating agricultural TFP, and then analyzed the spatial-temporal characteristics and spatial effects of GTFPI.

## 3. Results

### 3.1. Comparison of GTFPI and TFPI

During the sample period, GTFPI was substantially higher than the TFPI. The gap between GTFPI and TFPI was volatile, and it is worth noting that it increased significantly in 2015 and 2016. During the entire sample period, the average annual growth rate of GTFP was 4.07%, and the TFP was only 3.03%.

**Figure 2:**
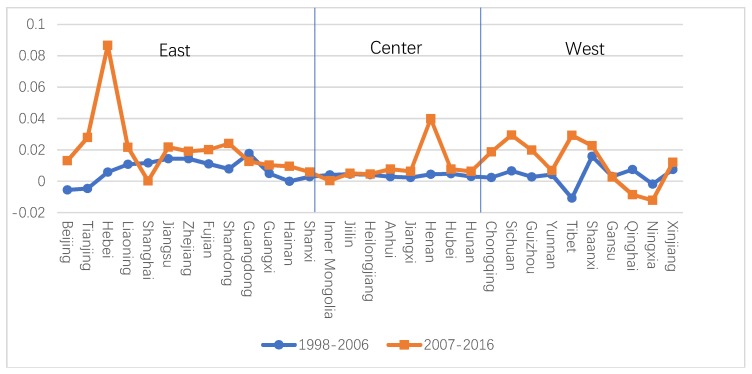
The mean gap between green total factor productivity index (GTFPI)and total factor productivity index (TFPI) in different periods in different provinces.

In order to better analyze the differences in the two indexes, this paper divided the sample period into two stages: 1998–2006 and 2007–2016, then compared them based on the three major regions—east, center, and west, as shown in Figure 2. The differences in GTFPI and TFPI in the three regions were nearly positive, and the average difference between them in 2007–2016 was mostly greater than that in 1998–2006. This indicated that GTFP had a higher growth rate, and this gap continued to widen in the second half of the period, which was the same conclusion as found in many previous studies. In the latter half of the period, the GTFPI in Hebei in the east, Hunan in the central area, and Tibet in the west were much larger than the TFPI. In contrast, the GTFPI in Shanghai and Ningxia were much smaller than the TFPI. We still compared the decomposition terms of the GTFPI and TFPI. It can be seen that the GTEC was smaller than the technology efficiency change (TEC) from 1997 to 2007 and larger than the TEC from 2008 to 2016, and the GTPC was greater than the technical progress change (TPC) at all times, but the gap widened further in the later time period.

**Figure 3:**
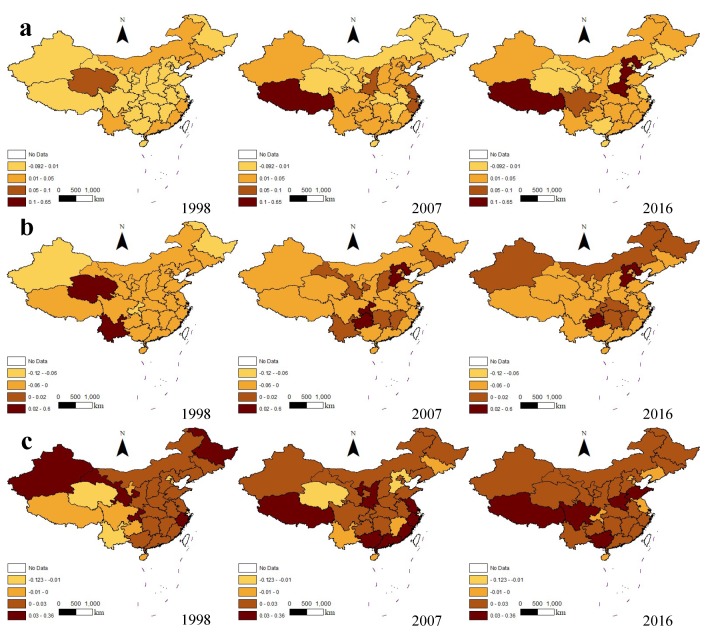
The difference values between the GTFPI and TFPI, GTEC and TEC, and GTPC and TPC in 1998, 2007 and 2016 were distributed in provinces across the country. (**a**) The gap between the GTFPI and TFPI; (**b**) the gap between the GTEC and TEC; (**c**) the gap between the GTPC and TPC.

Figure 3 shows the difference values between the GTFPI and TFPI, GTEC and TEC, and GTPC and TPC in 1998, 2007, and 2016 in 31 provinces nationwide. First, the dark areas in Figure 2a gradually increased, representing the difference between the GTFPI and TFPI increasing with time, which was the same as found in the analysis above. Many provinces were in the −0.092 to 0.01 stage in 1998, among which the GTFPI of Beijing, Shanghai, Sichuan and Guizhou was lower than the TFPI, and in 2007, the gaps of the four provinces became positive. In 2016, only Qinghai’s GTFPI was lower than its TFPI. Among all the provinces, the gaps of Hebei, Henan and Tibet saw a big increase. For example, Hebei increased from 0.004 in 1998 to 0.649 in 2016, Henan from 0.003 in 1998 to 0.287 in 2016, and Tibet from 0 in 1998 to 0.356 in 2016. In Figure 2b, similarly, the dark areas gradually expanded over time. However, the gaps between the GTEC and TEC in most areas were still negative. In some provinces, the difference gradually narrowed to 0 and eventually turned positive, including Hebei, Heilongjiang, Jiangxi, Hubei, Hunan, Chongqing and Xinjiang. In Figure 2c, except Liaoning, Guangdong, Shanxi, Heilongjiang and Xinjiang, the overall difference between the GTPC and TPC increased gradually. It is worth noting that in 1998, areas with a smaller GTPC than TPC, such as Yunnan, Guizhou, Sichuan and Qinghai in southwest China, were transformed into areas with a greater GTPC than TPC in 2016. In 2016, only Chongqing had a negative balance, and the gap did not exist in Beijing, Tianjin, Liaoning, Shanghai, Jiangsu and Hainan; their GTPC was equal to their TPC.

### 3.2. Analysis of Temporal and Spatial Characteristics of China’s Agricultural GTFPI

In the sample cycle, China’s agricultural GTFP showed a trend of continuous growth, with an average annual growth of 3.5%. The GTEC was mostly below 1, indicating green efficiency having an overall downward trend, while the GTPC was only less than 1 in 2008, and more than 1 in other years. According to the decomposition results, the growth of China’s agricultural GTFP mainly depended on agricultural green technology progress (GTP), and its contribution rate reached 4.6%. The average GTEC was less than 1, offsetting some of the growth from green technological progress.

In this paper, the sample period was divided into three periods: 1998–2003, 2004–2009, and 2010–2016, shown in Table 4. In any time period, the GTFP in China and the eastern central and western regions of China showed an increasing trend. The GTEC was less than 1 in the whole sample period and nationwide, showing that green technology had a decreasing trend. It should be noted that green technical efficiency (GTE) increased on average between 2004 and 2009 in the east and decreased on average between 1998 and 2003 and between 2010 and 2016. However, GTE in the east gradually increased on average during the whole sample period. GTP increased in three time periods and all regions, which showed that the improvement of China’s agricultural GTFP was mainly driven by GTP.

Taking 1998 as the base period, GTFP, GTE and GTP were all set as 1. We calculated their cumulative value over the sample period. During the period 1998–2003, TFP increased only slightly. As GTE declined, GTP did not fully offset the negative effects, because although China’s agriculture had a high yield in that period, due to market problems and agricultural structural contradictions, the grain could not be sold, which seriously affected the enthusiasm of Chinese farmers in production. From 2004 to 2009 the GTFP grew slowly, with an average annual growth rate of 2.6% which was mainly because the Chinese government has introduced a number of reform policies for rural areas, including abolishing agricultural taxes and subsidizing farmers. From 2010 to 2016, the GTFP showed an annual growth rate of 7.2%. That was because during this period, the Chinese government continued to increase its expenditure on agriculture and vigorously promoted agricultural technology, increasing the role of agricultural technology in promoting GTFP. Throughout the sample period, GTE continued to decline, while GTP continued to increase. This phenomenon showed that the developed technology has not been well used in practice, has not formed a demonstration effect, and has not been widely converted into productivity.

The average agricultural GTFPI in each province was consistent with the national level throughout the sample cycle. Agricultural GTFP was increasing, GTE was decreasing, and GTP was increasing. The agricultural GTFP increase was mainly driven by agricultural GTP. However, there were some differences in the speed of agricultural GTFPI in eastern, central and western China, in that growth was fastest in the east, followed by the central region, and was slowest in the west. The GTFP increased by 4.2% annually in the east region, 3.4% annually in the center, and 2.5% annually in the west.

**Figure 4:**
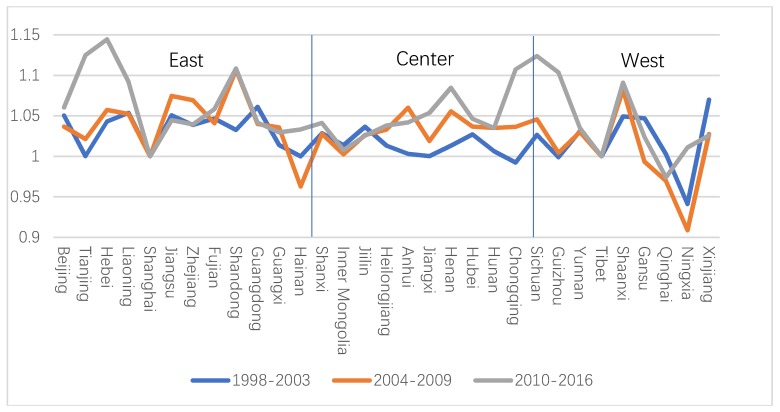
GTFPI in three periods in each province.

The study divided the sample period into three periods: 1998–2003, 2004–2009 and 2010–2016, as shown in Figure 4. From the three periods, the fluctuation trend of each sub-period was highly consistent, and the fluctuation range was different. In most provinces, the overall productivity growth rate was above 1 in each sub-period, realizing the growth effect. It is worth noting that Gansu, Qinghai and Ningxia in the western region showed negative growth during the period from 2010 to 2016.

**Figure 5:**
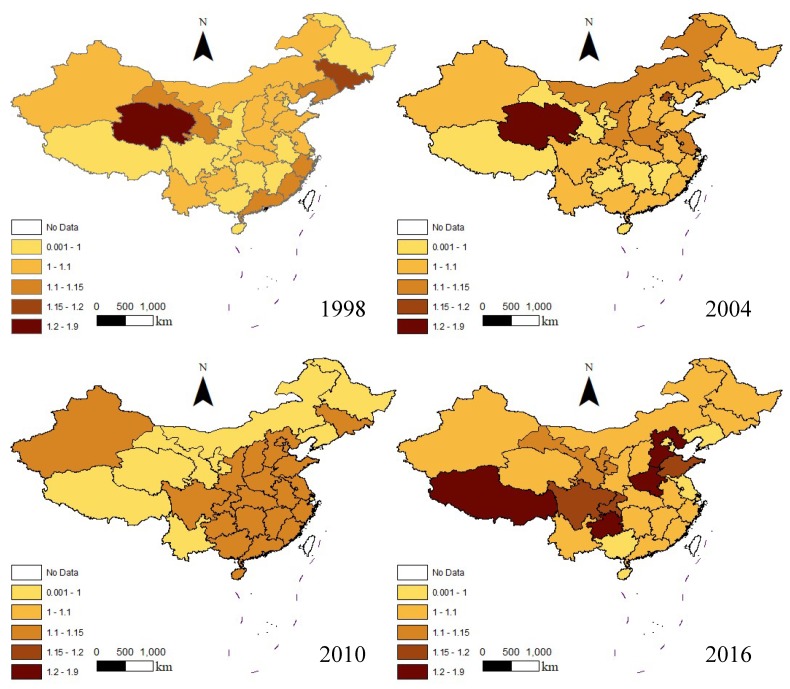
The spatial distribution of China’s agricultural GTFPI in 1997, 2004, 2010 and 2016.

In this paper, 1998, 2004, 2010 and 2016 were selected as observation points for the distribution maps of the GTFPI of various provinces in China, as shown in Figure 5. It can be seen from the figure that some areas have fluctuated greatly over time. Except for some provinces, the overall dark areas gradually expanded, and the GTFPI as a whole was in an incremental state. From the perspective of the spatial dimension, the spatial regional difference was relatively small in 1998, and expanded in 2016. It can be seen that in 2010, the trend of agricultural GTFPI accumulation was relatively obvious, but in the other three years, the high level agricultural GTFPI provinces did not exert an agglomeration effect.

By analyzing the temporal and spatial characteristics of the GTFPI and its decomposition terms, some main conclusions are obtained in this paper: GTE was in decline and GTP was increasing, promoting GTFP growth from 1998 to 2016; and China’s agricultural GTFP is the fastest in the east, followed by the center, and the slowest in the west. These findings were mostly in line with initial expectations. The emergence of the phenomenon of GTE being in decline and GTP increasing was because of a scientific research supply and actual demand disconnection, scientific and technological achievements being idle, and a low conversion rate. Investment in agricultural research and development and the extension of agricultural technology in China have been continuously promoted. However, the transformation of agricultural scientific research results is not timely, and the extension system of agricultural technology and the socialized service system of agriculture responsible for any transformation do not perform effectively.

### 3.3. The Spatial Correlation Analysis of the GTFPI

Agricultural TFP is determined by a variety of factors. A similar resource endowment and agricultural production environment would inevitably lead to similarity in agricultural GTFP. On the premise of such similarity, the cost of factor flow between neighboring provinces is low, which is beneficial to factor flow and technology diffusion and improved the spatial dependence between provinces. Through the above figure, it was found that GTFPI showed an agglomeration effect among provinces in 2010. Here, it was predicted that GTFPI would have a spatial interaction among provinces.

To test this hypothesis, this paper used the Moran index to measure the spatial correlation of the GTFPI. To ensure the reliability of the conclusion, the first-order adjacency weight matrix and the reciprocal square of distance were used to calculate the Moran index, as shown in Table 5. More than half of the years in which the indexes were calculated using the two weight matrices were not significant, and those years in which the index values were significant were also very small. Therefore, the hypothesis that GTFPI has spatial effects among provinces was not valid.

The results differed from those of many other studies. The first reason for this was that this paper adopted a more reasonable way to calculate ACEs, which was closer to reality. Taking ACEs as an undesired output, the measured GTFPI was naturally different from other studies. Second, compared with the DEA-Malmquist model and SFA model adopted in many studies, the SBM-GML model adopted in this paper made the calculation results of GTFPI more reasonable. Therefore, we believe that such results can better reflect China’s actual national conditions.

First, agriculture is a sector that uses natural resources as production objects, and its production depends largely on the natural endowments of the region. Admittedly, the development of neighboring areas can indirectly affect the agricultural production of the region. However, China has a vast territory and diverse topography. Climate conditions in various regions are also very different, and as this paper analyzed at the provincial level, there are big differences between provinces in resource endowment. Therefore, the neighboring areas have a limited influence, particularly in areas such as Sichuan and Tibet, Gansu, Qinghai, Yunnan, and other provinces bordering them. Due to climatic and geographical reasons, Sichuan is mainly engaged in traditional agriculture, while Tibet is more focused on animal husbandry. The crops cultivated in Gansu, Qinghai and other provinces are also very different from those grown in Sichuan, and the agricultural development situation among provinces is diverse. Therefore, their agricultural production is relatively independent. Second, Chinese provincial governments have the right to formulate a variety of agricultural development policies for their province. Accordingly, this has caused the development of agriculture in each province to be independent to a certain extent.

## 4. Discussion

Although there has been a lot of research on China’s agricultural green total factor productivity, there are still two points to be improved.

The first relates to undesired output. Most studies take agricultural carbon emissions as an undesired output, but when calculating agricultural carbon emissions, the selection of carbon emission sources is not fully considered. In the early days, a large proportion of research focused on agricultural material as the sole source of carbon emissions. With further development of this area of research, some scholars have added carbon emissions generated by rice to carbon emission sources, but still did not consider that the type and location of rice would also affect carbon emissions. Most scholars did not include soil N_2_O, as a source of carbon emissions, and those who did failed to take into account that crop varieties grown on the soil also affect N_2_O emissions. The studies that have looked into carbon emissions from livestock and poultry farming are not comprehensive enough in the selection of the species. Research measuring the agricultural GTFP has tended to focus on the measurement part, and there has not been enough consideration of the choice of ACESs. In recent years, there have been some relatively comprehensive studies, but these still have also omitted some important sources.

The second is the GTFP calculation method. In some studies, ACEs were taken as an input variable and GTFP was calculated with the general DEA model or SFA model. This was reasonable to some extent, but not rigorous enough. By contrast, the SBM directional distance function is the most effective method. However, the distance function fails to effectively deal with the inconsistency of the production frontier of production units in each period, which affects the comparability of inter-period results.

Therefore, this study intended to make breakthroughs in the above two aspects. Firstly, the paper would take the following carbon emission issues into account. The variety (early rice, late rice and in-season rice) and location of the rice affects its carbon emissions. In addition, the types of crops that grow in the soil would also influence the N_2_O emissions, and different types of livestock and poultry had different coefficients. Furthermore, straw combustion was included into the ACESs to ensure more accurate ACEs. Secondly, the paper adopted the GML index based on the SBM directional distance function to calculate GTFP, because it can not only deal with radial and angle problems causing the GML index to bias effectively, but also realize the global comparability of the production frontier.

Our research leads to the following main conclusions. Firstly, GTFP and TFP were both increasing annually, but the former was growing faster than the latter, and the difference widens later. The GTEC was smaller than the TEC at first, but the gap between them was narrowing with time, until the former became larger than the latter. The GTPC was bigger than the TPC, and the gap had widened over time. Secondly, GTE was in decline and GTP was increasing, promoting GTFP growth from 1997 to 2016. China’s agricultural GTFP increased by 4.2% annually in the east, 3.4% annually in the central region, and 2.5% annually in the west. Thirdly, there was no spatial correlation between China’s provinces in the GTFP index.

## 5. Conclusions

This paper measured agricultural carbon emissions in terms of five aspects—agricultural materials, rice planting, soil N_2_O, livestock and poultry farming, and straw burning—and then compared the green total factor productivity index and the total factor productivity index. There are still some deficiencies in this research, however. First, although the selection of ACESs in this paper is more comprehensive and in-depth, it can still only represent the real ACEs to an extent. Due to the progress of related natural sciences, there are still many ACEs that we cannot measure. Secondly, straw burning is taken as one of ACESs, which indeed promotes the research of ACEs measurement. Straw burning has been banned by some local governments in recent years, however, due to policy lag and lax supervision, the actual effect is not obvious, which is the reason for this paper including it in the ACESs. However, the actual quantity of straw burning in each province was not investigated on the spot in this study, so the quantity calculated will be different to the actual value.

## Figures and Tables

**Table 1:** Construction of input and output indicators.

Output	Desired Output	Actual Output Value of Agriculture, Forestry, Husbandry and Fishery (Based on 1997)
	Undesired Output	Agricultural Carbon Emissions
Input	Labor	Agricultural practitioners
	Land	Total area sown to crops
	Machinery	Total power of agricultural machinery
	Fertilizer	Application quantity of chemical fertilizer (refractive index)
	Pesticide	Consumption of chemical pesticides
	Agricultural film	Application amount of agricultural film
	Irrigation	Effective irrigation area
	Farm animals	The number of large cattle at the end of the year

**Table 2:** CH_4_ emission coefficients of different rice varieties in China’s provinces (unit: g/m^2^).

Area	ER	LR	IR	Area	ER	LR	IR	Area	ER	LR	IR
Beijing	0	0	13.23	Anhui	16.75	27.6	51.24	Sichuan	6.55	18.5	25.73
Tianjin	0	0	11.34	Fujian	7.74	52.6	43.47	Guizhou	5.1	21	22.05
Hebei	0	0	15.33	Jiangxi	15.47	45.8	65.42	Yunnan	2.38	7.6	7.25
Shaanxi	0	0	6.62	Shandong	0	0	21	Tibet	0	0	6.83
Inner Mongolia	0	0	8.93	Henan	0	0	17.85	Shaanxi	0	0	12.51
Liaoning	0	0	9.24	Hubei	17.51	39	58.17	Gansu	0	0	6.83
Jilin	0	0	5.57	Hunan	14.71	34.1	56.28	Qinghai	0	0	0
Heilongjiang	0	0	8.31	Guangdong	15.05	51.6	57.02	Ningxia	0	0	7.35
Shanghai	12.4	27.5	53.87	Guangxi	12.41	49.1	47.78	Xinjiang	0	0	10.5
Jiangsu	16.1	27.6	53.55	Hainan	13.43	49.4	52.29				
Zhejiang	14.4	34.5	57.96	Chongqing	6.55	18.5	25.73				

Note: ER = Early Rice, LR = Late Rice, IR = In-Season Rice.

**Table 3:** The carbon emission coefficients of major livestock (unit: kg/head/year).

Resources	CH_4_ Emission Coefficient		N_2_O Emission Coefficient
	Enteric Fermentation	Manure Management	
Cow	68	16	1
Buffalo	55	2	1.34
Cattle	47.8	1	1.39
Mule	10	0.9	1.39
Camel	46	1.92	1.39
Donkey	10	0.9	1.39
Horse	18	1.64	1.39
Live pig	1	3.5	0.53
Sheep	5	0.15	0.33
Goat	5	0.17	0.03
Rabbit	0.254	0.08	0.02
Poultry	-	0.02	0.02

**Table 4:** China’s agricultural GTFPI, GTEC, and GTPC in different periods and regions.

	Time	1998–2016	1998–2003	2004–2009	2010–2016
Whole	GEC	0.9898	0.9789	0.9972	0.9923
	GTC	1.0457	1.0440	1.0256	1.0635
	GTFPI	1.0348	1.0219	1.0228	1.0553
East	GEC	1.0006	0.9914	1.0125	0.9984
	GTC	1.0416	1.0413	1.0145	1.0656
	GTFPI	1.0422	1.0323	1.0271	1.0639
Center	GEC	0.9819	0.9581	0.9954	0.9912
	GTC	1.0532	1.0600	1.0375	1.0610
	GTFPI	1.0341	1.0156	1.0327	1.0516
West	GEC	0.9834	0.9829	0.9808	0.9860
	GTC	1.0425	1.0329	1.0285	1.0632
	GTFPI	1.0252	1.0153	1.0087	1.0483

**Table 5:** Global Moran index of the GTFPI from 1998 to 2016.

	TFP			
	W_q_		W_d_	
Year	MI	PV	MI	PV
1998	−0.097	0.261	−0.054	0.243
1999	−0.005	0.398	−0.067	0.146
2000	0.148	0.044	0.032	0.018
2001	−0.257	0.028	−0.082	0.077
2002	−0.018	0.440	−0.013	0.249
2003	0.017	0.320	−0.021	0.348
2004	−0.112	0.203	−0.088	0.026
2005	0.051	0.183	−0.061	0.155
2006	−0.056	0.387	−0.049	0.254
2007	0.230	0.012	0.085	0.000
2008	0.194	0.012	0.038	0.008
2009	0.002	0.364	−0.012	0.235
2010	0.086	0.112	0.006	0.085
2011	0.018	0.332	0.029	0.036
2012	−0.016	0.440	−0.042	0.390
2013	0.090	0.143	0.004	0.132
2014	0.216	0.011	0.015	0.067
2015	−0.109	0.162	−0.063	0.094
2016	−0.040	0.474	−0.021	0.353

Note: MI: Moran Index, PV = *p*-Value, W_q_: the first-order adjacent weight matrix, W_d_: the geographic distance matrix.
